# Loop-mediated isothermal amplification for the detection of goose circovirus

**DOI:** 10.1186/1743-422X-9-110

**Published:** 2012-06-13

**Authors:** Grzegorz Woźniakowski, Wojciech Kozdruń, Elżbieta Samorek-Salamonowicz

**Affiliations:** 1 Department of Poultry Viral Diseases, National Veterinary Research Institute, Partyzantów 57 Avenue, 24-100, Puławy, Poland

**Keywords:** Goose circovirus, Loop-mediated isothermal amplification, SYBR Green, GelRedTM stain solution

## Abstract

**Background:**

Goose circovirus (GCV) presents an immunosuppressive problem in production of geese. The infection’s clinical symptoms include growth retardation or feathering disorders but the infection process may remain non-symptomatic what makes the infected birds more susceptible for secondary viral, bacterial and fungal infections. Diagnosis of GCV infection is made by histopathological examination, dot blot hybridization, polymerase chain reaction (PCR) and real-time PCR. However these techniques require application of thermocyclers and qualified staff which may be cost-consuming for some diagnostic units. The aim of this study was to develop loop-mediated isothermal amplification assay (LAMP) as a simple method of GCV detection.

**Results:**

The presented study has shown LAMP as a rapid tool of detecting DNA of goose circovirus (GCV) as soon in 30 min time. The method used three sets of primers: two outer primers (F3 and B3), two inner primers (FIP and BIP) and two loop primers (FL and BL) to accelerate the reaction. The optimum reaction temperature and the time were 61°C for 30 min, respectively. The results were analysed using SYBR Green dye and GelRedTM solutions. Thirty-eight isolates of GCV collected from geese flocks in Poland were examined. For comparison, real-time polymerase chain reaction with F3 and B3 primers and SYBR Green dye was conducted. The obtained results have shown GCV-LAMP as a sensitive, rapid and specific assay and alternative for PCR-based methods.

**Conclusions:**

The developed technique due to its simplicity may be applied by any veterinary laboratory or even mobile diagnostics units for the routine detection of GCV.

## Background

Circoviruses represent a group of non-enveloped and small viruses about 20 nm in diameter with icosahedral symmetry [1]. Even though their history is not long, they present a serious immunosuppressive problem during infection of pigs, poultry, waterfowl and wild birds [1-5]. The *Circoviridae* family comprise of two genera: *Gyrovirus* which is represented by chicken anemia virus (CAV), one of the most economically serious but also well studied problem in poultry production and *Circovirus* genus represented by Pisstacine beak and feather disease virus (PBFDV), porcine circovirus types 1 and 2 (PCV1 and PCV2), which cause post-weaning multisystemic wasting syndrome (PMWS), pigeon circovirus (PiCV) detected for the first time in 1993, columbid circovirus (CoCV) also known as canary circovirus (CaCV), duck circovirus (DuCV), mute swan circovirus (SwCV) and goose circovirus (GoCV) [5-12].

Goose circovirus was described for the first time in 1998 [7] in a commercial geese flock showing runting and high mortality [13]. It is known that infection with GCV may be non-symptomatic facilitating secondary infection with other waterfowl pathogens as goose hemorrhagic polyomavirus (GHPV), goose parvovirus (GPV) or bacterial and fungal pathogens as *Riemerella anatipestifer* and *Aspergillus fumigatus*[1]. Geese affected with GCV may show growth retardation or feathering disorders. The most common histopathological changes are observed as depletion of T-lymphocytes and histocystis in lymphoid organs including thymus, spleen and bursa of Fabricius (BF) [14]. Diagnosis of GCV may help in isolation of the affected birds from the rest of the flock thus minimizing huge loss due to the GCV immunosuppressive influence.

So far the presented reports described isolation of GoCV in cell cultures that is laborious and needs SPF goose embryos, histology and electron microscopy which on the other hand require operation by an experienced pathologist [14]. The polymerase chain reaction (PCR) era of amplification of multiple genes and cloning of the whole DNA viral genomes facilitated the development of novel and sensitive techniques like nested-PCR and real-time PCR which allowed for firm and fast diagnosis as well as sequencing of circoviral genomes [13,15-19]. The other developed reliable diagnosis methods include dot blot hybrydisation (DBH) which was found to reflect the stadium and severity of GCV infection [13]. However application of these methods requires well-facilitated laboratories and experienced staff.

Due to these limitations during the last three years an increase in development of rapid and reliable loop-mediated isothermal amplification (LAMP) method has been observed.

LAMP is based on loop-amplification of a specific site of the gene by *Bst* or *Bsm* polymerases with DNA-strand displacement activity. Both enzymes originate from thermophile bacteria and catalyse 5´ → 3´ synthesis of DNA strand but lack 5´ → 3´ exonuclease activity. *Bst* polymerase is isolated from *Bacillus stearothermophilus* while *Bsm* originates from *Bacillus smithii*[20,21]. The idea of LAMP was described for the first time by Notomi et al*.*[20] who applied this technique for the hepatitis B virus detection. The method is sensitive at least as PCR and rapid which allows for a specific detection of different pathogens in less than 45 min. In short the method relies on DNA strand displacement and the following auto-cycling steps catalysed by *Bst* or *Bsm* polymerase in temperature of waterbath set between 60°C and 68°C [20-22]. LAMP uses two or three sets of primers complementary to six or eight different regions in the selected gene. The reaction uses forward inner and backward primer (FIP and BIP), outer forward and backward primer (F3 and B3) and forward and backward loop primers (FL and BL) which enable faster formation of loop and hairpin-like structures allowing faster detection of the specific DNA. The final products are known to be cauliflower-like structures with multiple overhangs and loops which can be observed during agarose gel separation of LAMP products. The direct evidence on the LAMP robustness is its application for the detection of many important poultry and waterfowl viral pathogens including West Nile Virus (WNV) [23], Marek’s disease virus (MDV) [22], goose parvovirus (GPV) [24], Muscovy duck parvovirus (MDPV) [25] and circoviruses comprising Porcine circovirus [26] and chicken anemia virus (CAV) [27]. The aim of this study was to develop and apply LAMP for the fast detection of 38 GCV field isolates from geese in Poland. This is the first report of the GCV detection by the new LAMP technique.

## Results

### Primer design

**Figure 1:**
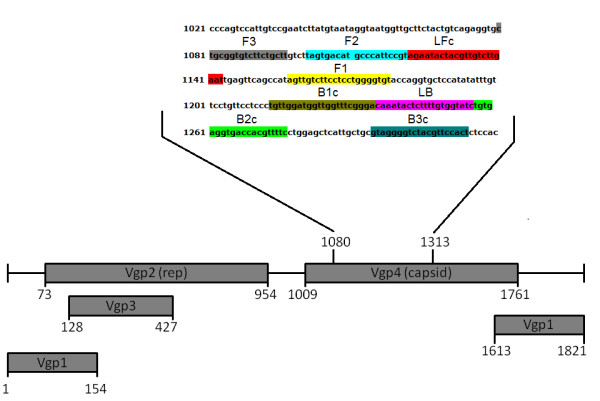
**GCV genome structure and localisation of designed LAMP primers in sequence of GCV (GenBank accession number NC_003054).** Descriptions: Vgp1 – viral protein 1, Vgp2 – viral protein 2 (rep – replication protein), Vgp3 – viral protein 3, Vgp4 – viral protein 4 (capsid protein), the numbers indicate the nucleotide position in the sequence, F3 – forward outer primer, F2 – first attachment site of FIP primer, LFc – attachment site of LF primer, F1 – second attachment site of FIP primer, B1c – first attachment site of BIP primer, LB – backward loop primer, B2c – second attachment site of BIP primer, B3c – attachment site for B3 primer, B3 – backward outer primer, FIP – forward inner primer, BIP – backward inner primer, LF – forward loop primer.

The designed three pairs of primers were located within the Vgp4 gene encoding GCV capsid protein (GenBank accession number NC_003054). The GCV genome encodes four viral proteins (Vgp1-Vgp4) within five opened reading frames (ORFs). The GCV genome structure, the primer sequences and their localisation in GCV genome were given in Table 1 and Figure 1.

**Table 1:** Sequences of LAMP primers used in the study

**Primer**	**Sequence 5’ – 3’**	**Length (nt)**	**Localisation in GCV genome**
F3	CTGCGGTGTCTTCTGCTT	18	1080-1097
B3	AGTGGAACGTAGACCCCTAC	20	1294-1313
BIP (B1c + B2)	TGTTGGATGGTTGGTTTCGGGA*TTTT*GAAAACGTGGTCACCTCACA	46	1214-1235, 1257-1276
FIP (F1c + F2)	ACACCCCAGGAGGAAGACAACT*TTTT*TAGTGACATGCCCATTCCGT	46	1157-1178, 1102-1121
LF	ATTCAAGACAACGTAGTATTCT	22	1122-1143
LB	CAAATACTCTTTTGTGGTATC	21	1236-1256

F3 – forward outer primer, B3 – forward backward primer, FIP – forward inner primer (F1c + F2), BIP – backward inner primer (B1c + B2), LF – forward loop primer, LB – backward loop primer, *TTTT* – thymine linker.

### LAMP optimisation

**Figure 2:**
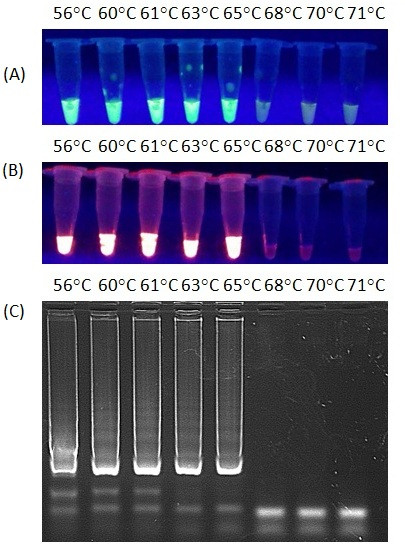
**Temperature gradient of LAMP.** The temperatures of the reaction mixtures were described above each tube. (**A**) The green fluorescence after addition of SYBR Green to each reaction mixture under UV light, (**B**) The orange fluorescence after addition of GelRed™ staining solution under UV light, (**C**) Electrophoresis of LAMP products in 2% agarose gel stained with GelRed™ DNA staining dye.

The optimal isothermal temperature was established as 61°C but the range from 56°C to 65°C had no influence on the results of LAMP (Figure 2A, B, C).

**Figure 3:**
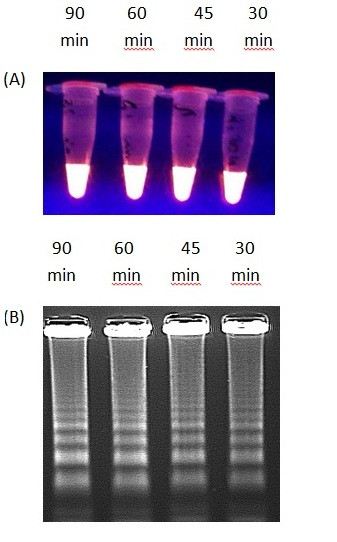
**LAMP. Incubation of the reaction mixtures for different time in the optimal reaction conditions.** (**A**) The orange fluorescence after addition of GelRed™ staining solution under UV light, (**B**) Electrophoresis of LAMP products in 2% agarose gel stained with GelRed™ DNA staining solution.

**Figure 4:**
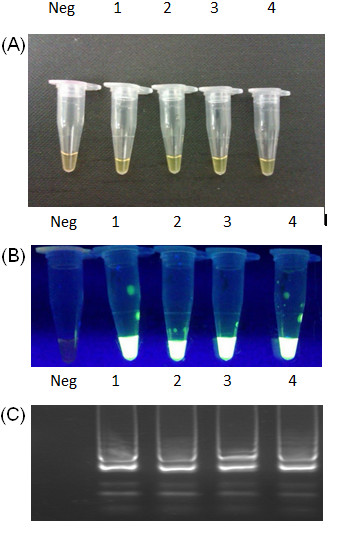
**Optimisation of *Bsm *polymerase concentration.** (**A**) Green color of LAMP positive samples, orange color of negative control, (**B**) The green fluorescence after addition of SYBR Green to each reaction mixture under UV light, (**C**) Electrophoresis of LAMP products in 2% agarose gel stained with GelRed™ DNA staining solution. Neg – negative control – reaction mixture without DNA template, 1 – *Bsm* polymerase 16 Units, 2 – *Bsm* Polymerase 8 Units, 3 – *Bsm* Polymerase 4 Units, 4 – *Bsm* Polymerase 2 Units.

**Figure 5:**
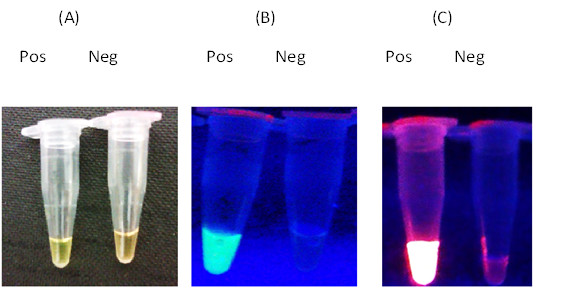
**Detection of LAMP product in (A) visible light, (B) fluorescence in UV after SYBR Green Addition, (C) fluorescence after GelRed**™ **addition.** Neg – negative control – reaction mixture without DNA template, Pos – DNA of P_1_03 strain.

The temperature above 68°C resulted in the reaction inhibition. The optimal LAMP time was determined to be 30 min, however the reaction underwent all the range of the examined time span up to 90 min (Figure 3A, B). The optimal primer concentration was set at 0.4 mM of each inner primer FIP and BIP, 0.1 mM of each outer primer F3 and B3, 0.2 of each LF and LR primer. However the reaction was flexible and allowed for the amplification of GCV DNA in all the examined primer concentrations. The *Bsm* polymerase concentration was set at 8 units (U). However amplification was possible using even the 2U of *Bsm* polymerase (Figure 4A, B). Both used dyes - SYBR Green as well as GelRedTM in concentration 1:10 of the stock solution were useful for visual determination of GCV DNA presence. However, more reliable results for naked-eye LAMP product detection were observed using SYBR Green which gave a clear orange color in negative samples and light green in positive ones. In the case of GelRedTM dye the positive samples turned pink while the negative remained red (Additional file 1). In UV light the strong orange signal was produced by GelRedTM stained LAMP samples but also green fluorescence given by SYBR Green was evident (Figure 5A, B, C).

### Sensitivity and specificity of LAMP and real-time PCR

**Figure 6:**
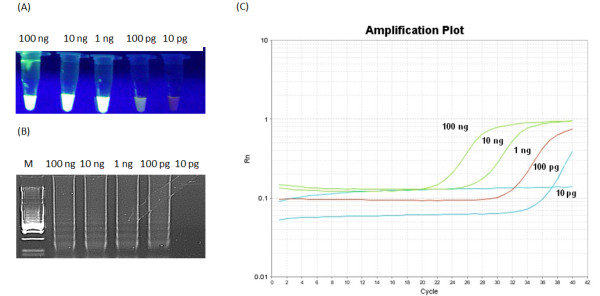
**Sensitivity of LAMP (A), (B) and real-time PCR for GCV detection.** Panel **(A)** shows green fluorescence after addition of SYBR Green to each reaction mixture under UV light, panel **(B)** electrophoresis of LAMP products in 2% agarose gel stained with GelRed™ DNA staining solution while panel **(C)** real-time PCR sensitivity plot.

**Figure 7:**
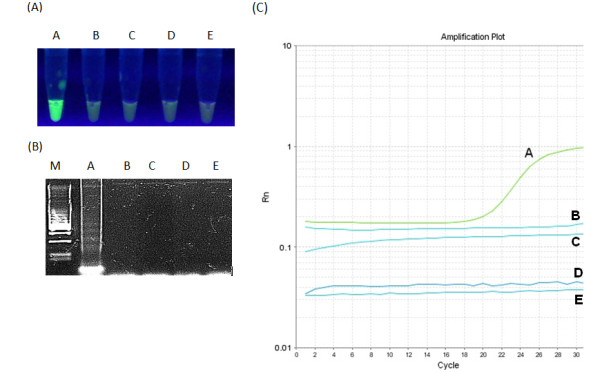
**Specificity of LAMP (A), (B) and real-time PCR for GCV detection.** Panel (**A**) shows green fluorescence after addition of SYBR Green to each reaction mixture under UV light, panel (**B**) electrophoresis of LAMP products in 2% agarose gel stained with GelRed™ DNA staining solution while panel (**C**) real-time PCR specificity plot. A - reference DNA of goose circovirus strain P_1_03, B - DNA of goose hemorrhagic polyomavirus (GHPV) strain 2003, C - Muscovy duck parvovirus strain FM, D - goose parvovirus strain B38, E - Fowl adenovirus type-1 strain CELO.

The detection limit of LAMP was 100 pg of DNA from P_1_03 strain (Figure 6A, B). In comparison real-time PCR detected the same amount of the reference strain DNA (Figure 6C). No positive signal was observed in DNA samples extracted from control samples of GHPV, GPV, MDPV, nor FadV-1 (Figure 7A, B, C). No bands were shown in negative controls after gel electrophoresis, indicating that LAMP facilitated rapid and specific detection of GCV.

### Field samples analysis by LAMP and real-time PCR

**Figure 8:**
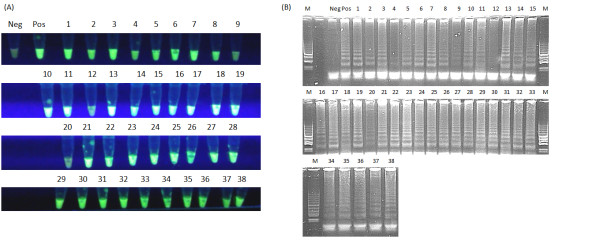
**LAMP. Analysis of 38 field samples from geese showing clinical symptoms.** Panel (**A**) shows green fluorescence after addition of SYBR Green to each reaction mixture under UV light, panel (**B**) electrophoresis of LAMP products in 2% agarose gel stained with GelRed™ DNA staining solution.

**Figure 9:**
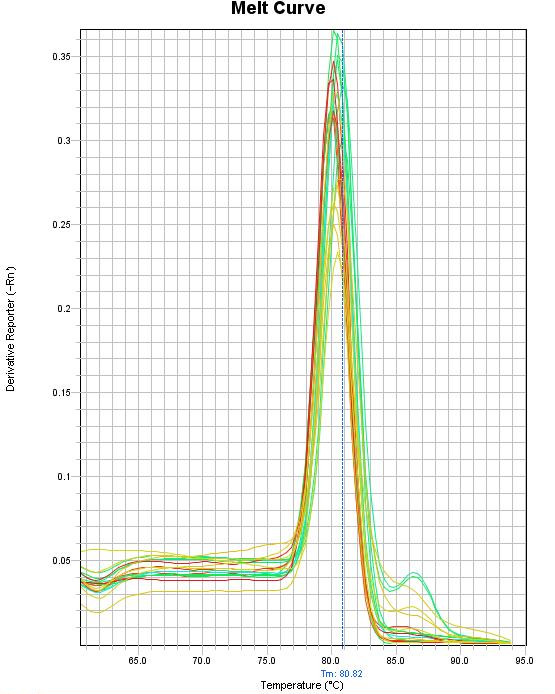
**The melting temperature analysis of GCV real-time PCR products.** The derivative reporter value is plotted as the y-axis while the temperature is plotted as the x-axis.

Total 38 different field samples collected from geese showing different clinical symptoms were tested (Figure 8A, B). Almost all samples (97.3%) except one (#12) were positive by LAMP. In two samples #4 and #20 the observed ladder-like pattern after gel electrophoresis was weak in comparison to their fluorescence. However the resluts retrived by real-time PCR confirmed data obtained from the LAMP assay (Table 2). The observed C_T_ values of these two samples reached 38.9 and 38.1 respectively. The positive C_T_ values were detected in geese showing clinical symptoms of GCV infection. The sample 12 was negative. The conducted analysis of the PCR products melting temperature revealed the same or very close temperature (79.9-80.8°C) for all the obtained products (Table 2; Figure 9). LAMP allowed for detection of GCV in waterbath for 30 min without thermocycler. There was no absolute need to separate reaction mixtures on gel since the observed fluorescence was specific and the gel electrophoresis was only performed to ensure the reliability of the designed assay.

**Table 2:** The cycle threshold and melting temperatures of GCV real-time PCR products

**Sample**	**Cycle treshold**	**Melting temperature °C**
Neg	40.0	-
Pos	11.4	80.2
1	31.7	80.0
2	36.7	79.9
3	31.1	80.0
4	38.9	80.1
5	29.3	79.9
6	31.7	80.0
7	35.8	80.2
8	28.6	79.9
9	37.9	80.2
10	36.8	80.0
11	35.9	80.0
12	40.0	-
13	32.0	80.0
14	36.1	80.0
15	34.6	80.2
16	30.9	80.2
17	34.6	80.4
18	32.0	80.6
19	21.6	80.2
20	38.1	80.3
21	30.7	80.4
22	36.4	80.2
23	19.3	80.4
24	34.2	80.4
25	35.9	80.4
26	35.6	80.4
27	34.2	80.2
28	31.2	80.2
29	20.4	80.2
30	27.4	79.9
31	26.7	80.4
32	27.1	80.4
33	29.2	80.6
34	37.0	80.6
35	26.1	80.6
36	33.7	80.8
37	36.3	80.4
38	30.6	80.8

The values of cycle threshold (C_T_) and melting temperature in °C were given in the table.

## Discussion

Circoviruses pose a serious threat for husbandry of all the economically important animals including pigs, poultry and waterfowl. Goose circovirus (GCV) represents a continuous and still not very well understood agent that causes immunosuppression in the affected waterfowl [1,9,26]. The first report of a clinical form of the disease occurrence with high mortality and stunting was described by Soike et al*.*[7]. The role of its immunity modulating activity was also presented in the context of the co-infection of geese with West Nile Virus in Hungarian geese flock [28]. An experimental approach for understanding the route of infection and pathology of the infection with circoviruses has recently been studied by Guo et al*.*[14] which confirmed and important role of these viruses in the clinical form of the disease manifested as severe feather disorders. However diagnosis drawn only on the basis of clinical symptoms themselves should also be confirmed by other techniques including histological assays, electron microscopy, serological assays, *in situ* hybridization [28] or different variants of polymerase chain reaction (PCR) [19]. Nowadays, PCR remains the most important tool in the detection of GCV and other circoviruses of birds. Recently a SYBR Green I-based real-time PCR has been applied for simple detection of duck circovirus (DuCV) in DNA extracted from different tissues of ducks [15]. The method allowed also for the quantification of DuCV in the investigated samples that facilitates also better understanding of the DuCV transmission and stage of the infection. Despite many advantages of PCR and real-time PCR these techniques show also limitations because they require PCR thermocyclers and even more complex real-time PCR systems that might not be affordable for small clinical units or mobile laboratories. The presented paper describes loop-mediated isothermal assay (LAMP) that allows simple, sensitive and specific detection of goose circovirus DNA with naked eye or under UV-light as soon as 30 min using only waterbath or dry block heating thermostat. LAMP is catalyzed by *Bst* or *Bsm* polymerase and two or three sets of primers complementary to eight fragments in the chosen gene. It has been previously shown that the technique presents a good alternative of PCR in detection of circoviruses including porcine circovirus type 2 [5] and chicken anemia virus (CAV) [26]. The presented GCV-LAMP was designed to facilitate detection of the virus in 30 min. In comparison the previously described LAMP techniques allowed for detection of circoviruses in around 60–90 min [5,22-24,26]. GCV-LAMP was specific only for GCV and no cross-reactive amplification with other viruses of waterfowl has been shown. The possible cross-reaction with DNA of pigeon circovirus (PiCV) was also excluded. The designed primers were complementary to Vgp4 gene sequence encoding capsid protein, what made them specific only for GCV. Using F3 and B3 primers in SYBR-Green based real-time PCR it was possible to confirm the results obtained by the new assay. However real-time PCR took over one and a half hour and required sophisticated apparatus. Moreover we have also successfully tested an alternative for widely-used SYBR-Green dye which is GelRedTM dye that gave strong fluorescence in UV light but was not useful for visual detection of GCV with naked eye. Concluding this study the developed GCV-LAMP technique allowed for robust and simple detection of goose circovirus in goose infections. The assay may be used by non-experienced laboratory staff using simple water bath and alternatively UV lamp.

## Methods

### DNA of reference and control viruses

The reference DNA of goose circovirus strain P_1_03 (Table 3) used in this study was a kind gift from dr Vilmos Palya (CEVA-Phylaxia, Ceva Sante Animale, Budapest, Hungary). The strain was used as the positive control for the developed real-time PCR SYBR Green assay and loop-mediated amplification. Negative controls used for testing specificity of real-time PCR and LAMP were: DNA of goose hemorrhagic polyomavirus (GHPV) strain 2003, Muscovy duck parvovirus strain FM (Vilmos Palya, CEVA-Phylaxia, Ceva Sante Animale, Budapest, Hungary), goose parvovirus strain B38, and Fowl adenovirus type-1 (FadV-1) strain CELO from the collection of the Department of Poultry Viral Diseases of National Veterinary Research Institute, Puławy, Poland.

**Table 3:** Accession numbers of reference and 8 field strains of GCV used in this study

**Strain**	**Accession number**	**Source**
P_1_03	JQ269340	CEVA-Phylaxia, Ceva Sante Animale, Budapest, Hungary
P_2_03	JQ269341	Field isolate, geese
P_3_03	JQ269342	Field isolate, geese
P_6_05	JQ269343	Field isolate, geese
P_10_05	JQ269344	Field isolate, geese
P_11_05	JQ269345	Field isolate, geese
P_13_07	JQ269346	Field isolate, geese
P_20_08	JQ269347	Field isolate, geese
P_50_08	JQ269348	Field isolate, geese

### Field isolates of GCV

Thirty eight field isolates of GCV were collected during the years 2003–2010 from sections of liver tissue from Polish flocks of graylag geese (*Anser anser*) showing different clinical symptoms of the virus infection. The geese were sent to the Department of Poultry Viral Diseases at the National Veterinary Research Institute, Pulawy, Poland for diagnosis of the disease. The 20 mg sections of liver from the examined birds were homogenized in 1 mL of polisaline phosphate buffer (PBS) with 1% additive of antibiotics (Antibiotic-antimitotic, Gibco) and subjected to DNA extraction. Accession numbers of partial nucleotide sequence of eight of these isolates were given in Table 3.

### DNA extraction

Total DNA was extracted from the homogenates of goose liver according to the procedure of the manufacturer of QIAamp DNA Mini Kit (Qiagen, Hilden, Germany). Briefly, 200 μL of homogenates were incubated with 20 μL of Proteinase K solution and AL buffer (Qiagen, Hilden, Germany) in 56°C for 10 min. After incubation to each solution 200 μL of ethanol was added and mixed vigorously. The solutions were centrifuged at 6000 × g (Mikro 22R, Hettich Zetrifugen, Tuttlingen, Germany) in silica-gel-based microcolumns (Qiagen, Hilden, Germany). The columns were then purified using AW1 and AW2 buffers and centrifugation according to recommendations of the manufacturer. The DNA was extracted with 100 μL of AE buffer (10 mM Tris·Cl; 0.5 mM EDTA, pH 9.0) supplied with the kit. The DNA were then frozen and stored in −70°C for further examination.

### Optimisation of loop-mediated amplification (LAMP) of GCV DNA

Primers for GCV-LAMP were designed according to the sequence of the complete sequence of goose circovirus (GenBank accession number NC_003054), using Primer Explorer version 4 (NetLaboratory, Tokyo, Japan). The forward inner primer (FIP) was designed based on the region F1c complementary to the F1 sequence, a TTTT-linker and the region complementary to F2 sequence. Similarly, BIP consisted of the B1c sequence complementary to B1, a TTTT-linker and B2 sequence. To accelerate the reaction loop-forward primer and loop-reversed primers were also designed and applied in the reaction. All reactions were set up on ice.

The LAMP reaction was carried out on in 25 μL containing: 1× Bsm Pol buffer (20 mM Tris–HCl (pH 8.8 at 25°C), 10 mM KCl, 10 mM (NH_4_)_2_SO_4_, 5 mM MgSO_4_, 0.1% Tween 20) (Thermo Scientific-Fermentas, Vilnus, Lithuania), 0.8 M betaine (Sigma-Aldrich, St Louis, Missouri, United States), 1.2 mM of each dNTPs (EurX), 0.4-1.2 mM each of inner primers FIP and BIP, 0.1-0.4 mM each of outer primers F3 and B3, 0.2-0.6 mM of each LF and LR primers, 2 - 8U *Bsm* DNA polymerase (Thermo Scientific-Fermentas, Vilnus, Lithuania), 1 μL of DNA template (~25 ng) and deionised water. Tubes were then incubated in a water bath (56 – 71°C) for times ranging from 30 to 90 min. The mixtures were then heated to 80°C for 10 min. After this step 1 μl of 1:10 stock dilution of 10 000 X concentrated in DMSO SYBR Green (Invitrogen) was added to each sample.Parallelly, for testing purposes 1 μL of 1:10 diluted 10 000 X in DMSO concentrated GelRedTM staining dye (Biotum, Delhi, India) was used. Samples were observed with naked eye on black background and under UV illumination (Bio-rad, 1000 Alfred Nobel Drive Hercules, California, United States) to observe color change during LAMP. Tubes were photographed with Wildfire S built-in digital camera (HTC, Bellevue Washington, United States). After reaction 10 μL LAMP products were separated under 120 V for 40 min in 2% agarose gels stained with 3 μL of GelRedTM staining dye (Biotum, Delhi, India). The specific ladder-like patterns of LAMP products were visualized and photographed under UV light in GenoSmart System (VWR, Randor, Pennsylvania, United States).

### Real-time PCR

Real-time PCR was run on ABI 7500 (Applied Biosystems, Foster City, California United States) with operating software in 2.0.1 version. The reactions were set up on in 0.2 ml OptiAmp® optical tubes with caps (Applied Biosystems, Foster City, California, United States) using Quantitect SYBR Green PCR Kit (Qiagen, Hilden, Germany) with application of outer primers F3 and B3 designed for LAMP. The reaction conditions were as follows: 25 μL of total mixture, 12.5 μl of 2x QuantiTect SYBR Green PCR Master Mix, 20 pmol of each F3 and B3 primer, 1 μL of DNA template (~25 ng) which was used in LAMP and deionised water.

### Comparison of LAMP and real-time PCR

To compare the sensitivity of LAMP to real-time PCR, 5 ten-fold dilutions (100 ng – 10 pg) of DNA of P_1_03 strain (200 ng/μL) were used as templates for each reaction. Specificity test was done using reference DNA of goose circovirus strain P_1_03 (CEVA-Phylaxia, Ceva Sante Animale, Budapest, Hungary) and set of negative controls: DNA of goose hemorrhagic polyomavirus (GHPV) strain 2003, Muscovy duck parvovirus strain FM (Vilmos Palya, CEVA-Phylaxia, Ceva Sante Animale, Budapest, Hungary), goose parvovirus strain B38, and Fowl adenovirus type-1 strain CELO from the collection from the Department of Poultry Viral Diseases of National Veterinary Research Institute, Puławy, Poland.

## Competing interests

The authors declare that they have no competing interests.

## Authors' contributions

Most of the experiments were conducted by GW who developed and optimised LAMP and real-time PCR assays. WK provided reference viruses and field isolates of GCV. ESS assisted in experimental design of the study. ESS and WK participated in the coordination of the study. GW wrote this manuscript and prepared figures for publication. The final manuscript was read and approved by all the authors.

## Supplementary Material

Additional file 1 **Figure 11.** GCV-LAMP. Samples after LAMP stained with 1μL of GelRedTM solution 1:10. Neg – negative control – reaction mixture without DNA template, Pos – DNA of P_1_03 strain. Click here for file
