# Supplementary material for: Loop-mediated isothermal amplification for the detection of goose circovirus
Source: Virol J. 2012 Jun 13;9:110. doi: 10.1186/1743-422X-9-110 (PMC3494565; doi:10.1186/1743-422X-9-110)

Additional Figure 11. GCV-LAMP. Samples after LAMP stained with 1µL of GelRed™ solution 1:10. Neg – negative control – reaction mixture without DNA template, Pos – DNA of P_1_03 strain.

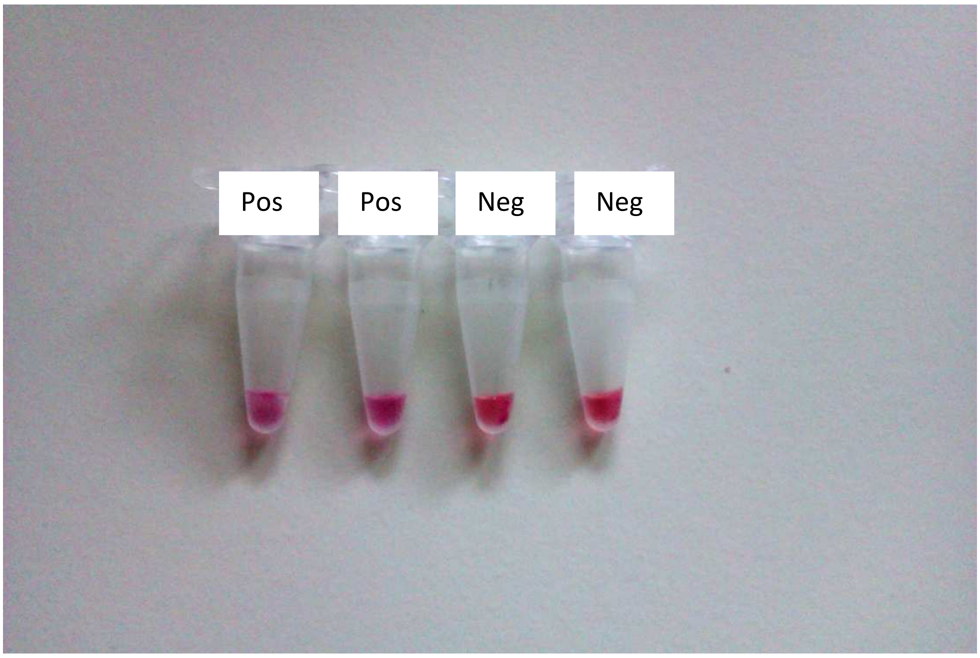

Supplement: Additional file 1 — Figure 11. GCV-LAMP. Samples after LAMP stained with 1μL of GelRedTM solution 1:10. Neg – negative control – reaction mixture without DNA template, Pos – DNA of P_1_03 strain. [file 1743-422X-9-110-S1.pdf]
